# The Effects of Glucagon‐Like Peptide‐1 Receptor Agonists on Mitochondrial Function Within Skeletal Muscle: A Systematic Review

**DOI:** 10.1002/jcsm.13677

**Published:** 2025-01-16

**Authors:** Victoria J. Old, Melanie J. Davies, Dimitris Papamargaritis, Pratik Choudhary, Emma L. Watson

**Affiliations:** ^1^ Department of Cardiovascular Sciences, College of Life Sciences University of Leicester Leicester UK; ^2^ Diabetes Research Centre, College of Life Sciences University of Leicester Leicester UK

**Keywords:** GLP‐1 RA, mitochondria, obesity, skeletal muscle

## Abstract

**Background:**

Obesity is a chronic disease associated with increased risk of multiple metabolic and mental health–related comorbidities. Recent advances in obesity pharmacotherapy, particularly with glucagon‐like peptide‐1 (GLP‐1) receptor agonists (RAs), have the potential to transform obesity and type 2 diabetes mellitus (T2DM) care by promoting marked weight loss, improving glycaemic control and addressing multiple obesity‐related comorbidities, with added cardio‐renal benefits. Dual agonists combining GLP‐1 with other enteropancreatic hormones such as glucose‐dependent insulinotropic polypeptide (GIP) have also been developed in recent years, leading to greater weight loss than using GLP‐1 RAs alone. However, up to 40% of the weight lost with GLP‐1 RAs comes from lean body mass, raising concerns about potential adverse effects on skeletal muscle function. Mitochondrial dysfunction, characterized by reduced mitochondrial size and activity, is prevalent in individuals with obesity and T2DM and is a known contributor to muscle wasting in ageing and some chronic diseases. This systematic review investigates the impact of GLP‐1‐based therapies on skeletal muscle mitochondrial function in individuals with obesity and T2DM or in related animal and cell models.

**Methods:**

A comprehensive search of MEDLINE, Scopus, CINAHL and clinicaltrials.gov was conducted. Inclusion criteria included randomized controlled trials, randomized crossover trials, cluster randomized control trials and basic science studies involving any GLP‐1 RA or GLP‐1/GIP dual agonist. Outcomes of interest were skeletal muscle respiratory function either in the form of measurements of mass, number, content, oxidative capacity/respiratory function, mitochondrial dynamics, mitochondrial biogenesis and mitophagy.

**Results:**

Eight studies were eligible for analysis; no human studies were identified. All of the included studies used GLP‐1 RAs (single agonists) as intervention. The emerging evidence suggests that GLP‐1 RAs increase mitochondrial area, number and morphology (i.e., reduces swelling). Data are conflicting on the effect of GLP‐1 RAs upon mitochondrial mass, respiration and the expression of uncoupling proteins and PGC‐1α. Data also demonstrate muscle specific (i.e., soleus vs. extensor digitorum longus) responses to GLP‐1 RAs.

**Conclusion:**

GLP‐1 RAs appear to have a positive effect upon mitochondria area, number and morphology, but effects upon other aspects of mitochondrial health remain inconclusive. Data are very limited and solely presented in animal and in vitro models. Future studies should be conducted in human populations in order to begin to understand the effect of GLP‐1 RAs and GLP‐1‐based therapies on human skeletal muscle mitochondria.

## 1 Introduction

Obesity is recognized as a chronic, progressive and relapsing disease process, which can lead to multiple comorbidities including type 2 diabetes mellitus (T2DM), obstructive sleep apnoea, metabolic dysfunction‐associated steatosis liver disease, hypertension, cardiovascular disease, and certain cancers [1]. T2DM affects 6.3% of the world population and is the ninth largest cause of mortality around the world [2]. Obesity plays a major role in T2DM development as it causes insulin resistance, a key pathophysiological process for T2DM. Considering the close relationship between obesity and T2DM, weight loss can result in beneficial effects on glycaemia, insulin resistance and cardiometabolic complications [3]. Treatment usually constitutes lifestyle interventions, bariatric surgery and pharmacotherapy [4].

The recent advances in glucagon‐like peptide‐1 (GLP‐1) receptor agonists (RAs) have transformed the care of obesity and T2DM. Exenatide was the first GLP‐1 RA approved by the food and drug administration (FDA) in 2005 [5] for the treatment of T2DM. Since then, the field has developed rapidly with many GLP‐1 RA approved for T2DM management and obesity. Recent years have seen a sharp rise in the prescription of weight loss drugs in general with prescription rates increasing by 114% between 2016 and 2021 [6].

More specifically, the efficacy of the injectable GLP‐1 RAs semaglutide 1 mg once weekly and liraglutide 1.8 mg once daily in inducing weight loss (WL) and weight maintenance in people with T2DM has led to clinical trials assessing higher doses of these molecules as treatments for obesity. Both semaglutide 2.4 mg once weekly and liraglutide 3 mg once daily have now received approval for chronic weight management. In clinical trials, liraglutide 3 mg in combination with a moderate intensity lifestyle intervention led to mean weight loss 6%–8% weight loss at 56 weeks compared with 2%–2.6% weight loss with placebo [7, 8]. On the other hand, the use of semaglutide 2.4 mg once weekly in combination with a moderate intensity lifestyle intervention resulted in 9.6%–14.9% mean weight loss after 68 weeks compared with 2.4%–3.4% weight loss in the placebo group [9, 10].

In recent years, the combination of GLP‐1 with other entero‐pancreatic hormones such as glucose‐dependent insulinotropic polypeptide (GIP), glucagon and amylin has been trialled (as dual and triple receptor agonists), holding the potential for even greater weight loss and glucose‐lowering effect than GLP‐1 RAs alone [11]. Tirzepatide, the first dual agonist approved for T2D and obesity management, acts on both GLP‐1 and GIP receptors and can lead to 15.6%–20.9% mean weight loss after 72 weeks of treatment [12, 13].

GLP‐1 RAs and dual GLP‐1/GIP agonists offer also many health benefits beyond weight loss and improved glycaemia. In people with T2DM and established (or high risk for) cardiovascular disease, multiple large cardiovascular outcome trials have shown that GLP‐1 RAs reduce the risk of major cardiovascular events (a composite outcome of cardiovascular death, non‐fatal stroke and non‐fatal myocardial infarction) [14]. In T2DM with chronic kidney disease, the FLOW trial demonstrated that semaglutide 1 mg lowers the risk of clinically important kidney outcomes and death from cardiovascular causes [15]. Moreover, in people with obesity (without diabetes) and established cardiovascular disease, the SELECT trial demonstrated that semaglutide 2.4 mg reduces by 20% the risk of major cardiovascular events compared with placebo [16]. Both GLP‐1 RAs and tirzepatide improve multiple cardiometabolic risk factors (waist circumference, systolic blood pressure, lipid profile) and quality of life parameters in people with obesity and/or diabetes [17]. Semaglutide 2.4 mg have also shown improvements in quality of life, physical limitations and exercise function in people with obesity and heart failure with preserved ejection fraction (HFpEF) [18, 19]. Additionally, tirzepatide significantly improves obstructive sleep apnoea (OSA) severity in people with moderate to severe OSA [19], liver inflammation and fibrosis in those with metabolic‐dysfunction–associated steatohepatitis and moderate to severe liver fibrosis [20] as well as heart failure outcomes in people with obesity and HFpEF. Early‐phase clinical trials as well as observational studies also suggest potential benefits of GLP‐1 RAs in people with Alzheimer's disease and those with Parkinson's disease (due to potential neuroprotective effects of GLP‐1 RAs) [21, 22, 23], when retrospective observational data suggest that GLP‐1 RAs may also reduce the risk for certain obesity‐related cancers such as colorectal, pancreatic and breast cancer [24].

Despite the marked weight loss and the important health benefits with GLP‐1 based therapies, the overall weight loss during these trials does not solely consist of fat mass. Emerging evidence shows that up to 40% of overall weight loss with semaglutide 2.4 mg, and up to 25% with tirzepatide, is lean body mass, which raises concerns that the drug therapy may hold a negative effect upon skeletal muscle mass and function, especially in populations at high risk of sarcopenia [25]. These losses could potentially have a negative impact people's day‐to‐day life, increase the risk of falls and decrease engagement in activities of daily living [26], whilst further exacerbating metabolic dysfunction [27]. Moreover, weight loss is also associated with bone loss, and concerns also lie with the effect of GLP‐1 therapy upon bone health. A randomized controlled trial showed that in people with obesity (without diabetes), 1 year of liraglutide 3 mg led to reduced hip and spine bone mineral density, an affect that could be offset by exercise [28]. On the other hand, a recent systematic review concluded a neutral impact of GLP‐1 RAs on bone mineral density [29]. Although outside the scope of the current review, this is an area requiring further attention.

Mitochondria are key organelles involved in energy production. Impairments in skeletal muscle mitochondrial function and content are common in obesity [30] and T2DM [31]. Dysfunctional mitochondria are important sources of reactive oxygen species (ROS) implicated in pathophysiological conditions including ageing and cardiovascular disease. Declines in mitochondrial function/abundance also appear to have direct effects on muscle wasting and are thought to be a principal driver of muscle wasting with age and in chronic disease [32, 33]. It has been demonstrated that mitochondrial dysfunction (including reductions in mitochondria size and citrate synthase activity) within skeletal muscle is already present in people with T2DM and obesity compared with lean controls [34]. With this in mind, investigating the effects of GLP‐1 RA–based therapies on mitochondrial function is important to fully understand the effect of this class of drugs upon skeletal muscle and for the potential design of adjunct therapies to offset any negative effects.

The aim of this systematic review was to systematically analyse existing literature on the effect of GLP‐1 RA–based therapies on skeletal muscle mitochondrial function within people with obesity or T2DM or relevant disease animal or cell models of obesity or T2DM.

## 2 Methods

The protocol has been registered on International Prospective Register of Systematic Reviews (PROSPERO) with the identifier CRD42024514415.

### 2.1 Search Strategy

Searches were conducted to identify any relevant studies using the following databases: MEDLINE, Scopus, CINAHL and clinicaltrials.gov. Databases were searched from inception to 22^nd^ of February 2024. Because of the difficulty in translation, only studies in English were included. In addition, these searches were supplemented with internet searches (e.g., Google Scholar) and further screening of reference lists of accepted papers.

### 2.2 Search Criteria

Inclusion criteria were as follows: (1) either randomized controlled trials, randomized crossover trials, cluster randomized control trials and basic science experimental trials; (2) studies using rodent animal model, in vitro models or human samples within the context of obesity with or without diabetes; (3) studies that investigated the use of GLP‐1 RA therapy with or without the combination of GIP as a pharmacological intervention with a comparison model of either no GLP‐1 RA therapy, use of a placebo or usual care; (4) primary outcome was skeletal muscle mitochondrial function either in the form of mitochondrial mass (measured by citrate synthase activity, polymerase chain reaction [PCR], western blotting or transmission electron microscopy [TEM]), oxidative capacity/mitochondrial function (by respirometry, western blotting or PCR), mitochondrial dynamics (by western blotting, PCR or TEM), mitochondrial biogenesis (by western blotting or PCR) and mitophagy (by western blotting or PCR); (5) full‐text published in English.

Exclusion criteria were as follows: (1) non‐English papers; (2) not skeletal muscle focused; (3) not mitochondrial related; (4) review articles.

### 2.3 Selection of Studies

Two independent reviewers conducted the study selection (VO, EW), based on the inclusion and exclusion criteria. The initial screening excluded duplicate papers and irrelevant papers. The remaining studies were reviewed with full texts screened against the inclusion/exclusion criteria. Any conflicts were resolved via discussion. The Cochrane flow diagram displays the selection process [35].

### 2.4 Data Extraction and Quality Appraisal

Data were extracted by VO using a standardized form and cross‐checked by EW. The following data were extracted: general study information, type of model used, cell line, GLP‐1 RA drug tested and comparator intervention, length of time of the intervention and outcome data. Studies were assessed for quality, eligibility and bias by one investigator (VO) using the Cochrane Risk of Bias Tool across five domains.

### 2.5 Data Analysis

The studies included in this review involved different methodologies and animal models. A meta‐analysis was not suitable because of the wide differences between outcome measures recorded and techniques used to assess. A qualitative review was performed.

## 3 Results

### 3.1 Characteristics of Included Papers

**FIGURE 1:**
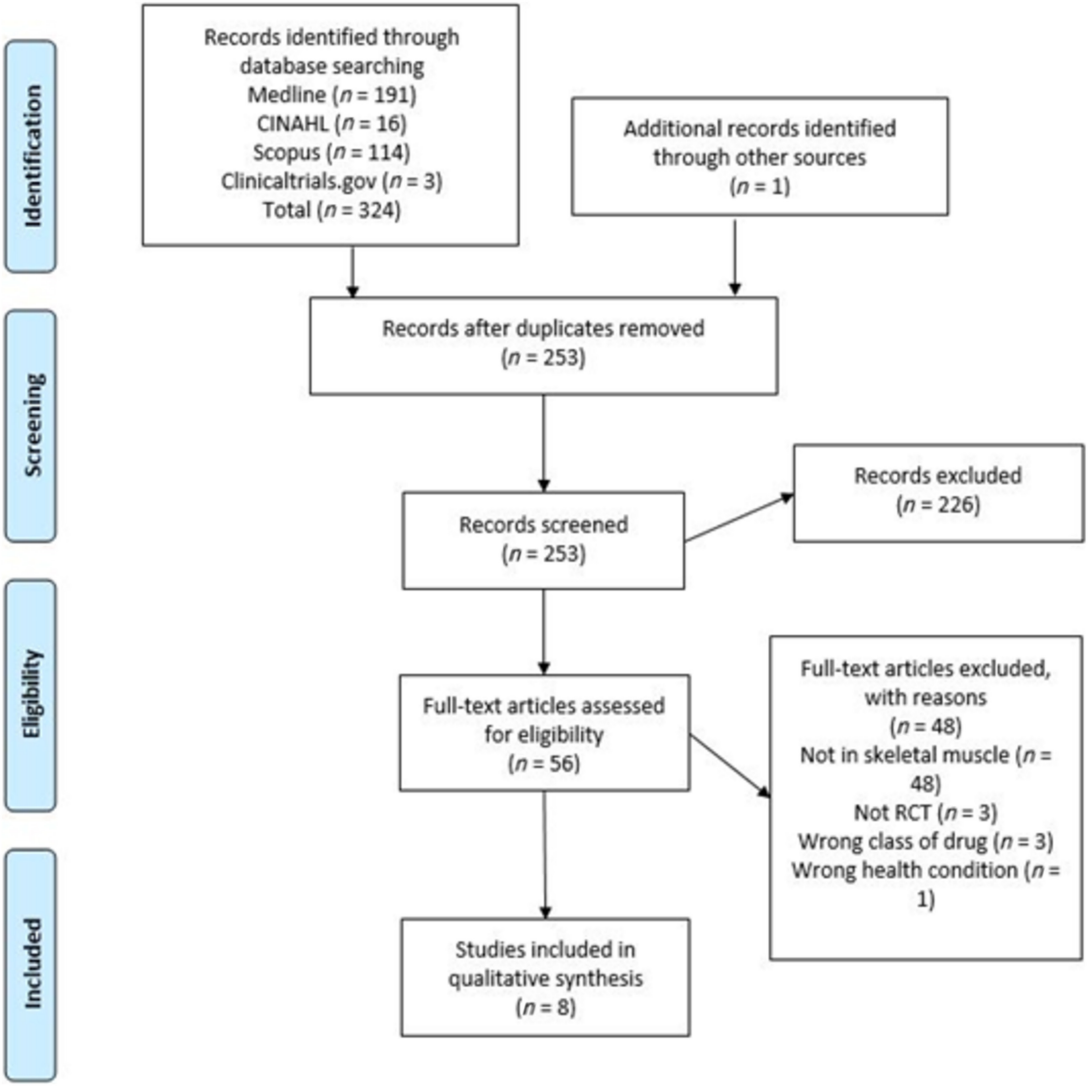
Prisma flow diagram of trial selection. Abbreviation: RCT, randomized controlled trial.

This review encompassed a total of eight studies; the study selection flow diagram can be found in Figure 1. Only one study used semaglutide [36], three used liraglutide [37, 38, 39], two used exenatide [40, 41] and two used Exendin‐4 [42, 43]. No studies using GLP‐1 RA GIP were found. All studies included were preclinical trials; no human studies were found. Of these, two trials were conducted using rat models, spontaneously diabetic torii rats (SDT‐rats) [37] or Sprague Dawley rats [40]; five trials used mice, including the C57BL/6J mouse line [36, 39, 42], KKAy mice (a cross breed between diabetic KK mice and lethal yellow Ay mice that carry a mutation of the agouti gene) and C57BL/6J mice were used by [38] and C57bL/6JOlaHSD mice (a subline of mice carrying a deletion of the alpha synuclein locus gene) were used by [41]. One in vitro study investigated GLP‐1 RA therapy using the C2C12 cell line [43]. In terms of our primary outcome, three studies reported the effect of GLP‐1 RA on mitochondrial density, area or morphology [36, 38, 40]; one study examined mitochondrial content, focusing on citrate synthase activity [37]; oxygen consumption rate was explored in two studies following different GLP‐1 RA therapy [41, 43]; two studies explored effects of GLP‐1 RA on mitochondrial biogenesis [37, 39] and two on metabolism [42, 43]. Study characteristics details can be found in Table 1.

**TABLE 1:** Characteristics and results summary of included studies. Carnitine palmitoyltransferase I (CPT‐1), extensor digitorum longus (EDL), high‐fed diet (HFD), peroxisome proliferator–activated receptor gamma coactivator‐1 alpha (PGC‐1α), not reported (NR), spontaneously diabetic torii (SDT), type 2 diabetes mellitus (T2DM), normal control diet (NCD), high‐fed high sucrose (HFHS), uncoupling protein 1 (UCP1), uncoupling protein 2 (UPC2), uncoupling protein 3 (UPC3). *Strong trend towards an increase in STD + liraglutide versus STD control *p* = 0.05.

Trial	Type of Trial	Model	Sample size/experiment repeats	Drug therapy	Duration + dosage	Comparator	Mito‐related outcomes and direction of change
[36]	Preclinical	C57BL/6J mice	Control *n* = 6 HFD *n* = 6 HFD + Semaglutide *n* = 6	Semaglutide	12 weeks, 30 nmol/kg/day	Untreated HFD + untreated normal	Number ↑ + area ↑ HFD + semaglutide vs. untreated HFD
[37]	Preclinical	SDT rats	SD‐control *n* = 7 SDT‐control *n* = 7 SDT + liraglutide *n* = 6	Liraglutide	8 weeks of increased daily doses: Week 1—0.2 mg/kg, week 2—0.4 mg/kg, week 3–8—0.6 mg/kg	SD untreated + SDT untreated	Citrate synthase ↔ soleus* & EDL, Cox5B ↑ soleus & EDL PGC‐1α ↔ soleus, ↑ EDL
[40]	Preclinical	SD rats	T2DM‐control *n* = 5 T2DM + exenatide *n* = 5 Non‐T2DM‐control *n* = 6 Non‐T2DM + exenatide *n* = 6	Exenatide	8 weeks, daily dose of 5 μg	Untreated T2DM + untreated non‐T2DM	Swelling ↓in T2DM + exenatide vs. untreated T2DM
[42]	Preclinical	C57BL/6J mice	EGLP *n* = 4 Exendin‐4 *n* = 4 Control *n* = 4	Exendin‐4, EGLP‐1	10 weeks of twice daily dose. 30 nmol/kg	Untreated C57BL/6J	UCP3 ↑ in EGLP‐1 vs. Exendin‐4 & untreated, UCP1 ↔
[43]	Preclinical	C2C12 cell line	Repeats ‐ NA	Exendin‐4	20 nM changed every 24 h for 48 h total	Untreated C2C12	Basal OCR ↑ LEAK state ↑ maximum respiration ↑ uncoupling efficiency ↑ UCP1 mRNA↑ UCP2 mRNA ↔ UCP3 mRNA ↔ CPT‐1 mRNA ↔ PGC‐1α protein ↑
[41]	Preclinical	C57BL/6JOlaHSD mice	NCD *n* = 10 HFD + exenatide *n* = 10 HFD *n* = 10	Exenatide	8 weeks of 10 μg/kg/day	Normal diet untreated + HFD untreated	Mitochondrial respiration ↔ overall (LEAK state ↔ OXPHOS state ↔ electron transfer system capacity ↔)
[38]	Preclinical	KKAy and C57BL/6J mice	KKAy‐control *n* = 6 KKAy + liraglutide *n* = 6 C57 control *n* = 6	Liraglutide	6 weeks of 250 μg/kg/day	KKAy untreated + C57BL/6J untreated	Number ↑ in KKAy + liraglutide vs. KKAy untreated, area ↑ in KKAy + liraglutide vs. KKAy untreated
[39]	Preclinical	C57BL/6J mice	Normal diet control *n* = NR HFHS control *n* = NR Normal diet + liraglutide *n* = NR HFHS + liraglutide *n* = NR	Liraglutide	4 weeks of 0.1 mg/kg/day	Chow untreated + HFHS untreated	PGC‐1α ↑ in normal diet + liraglutide vs. normal diet control & ↑ in HFHS + liraglutide vs. HFHS control

### 3.2 Risk of Bias

**FIGURE 2:**
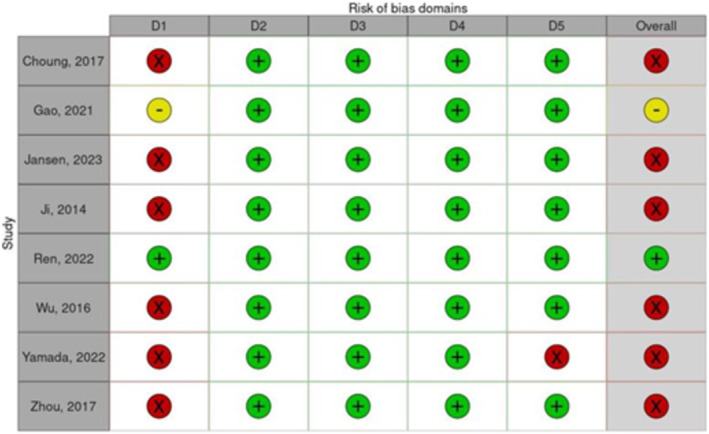
Cochrane Risk of bias tool used to assess the risk of bias in included trials. Judgements indicated as high risk of bias ‘X’, some concerns ‘‐’, low risk of bias ‘+’. Domains are as follows: bias arising from the randominization process (D1), bias due to deviations from intended intervention (D2), bias due to missing outcome data (D3), bias in measurement of the outcome (D4), bias in the selection of the reported result (D5).

Summaries of the studies' risk of bias are provided in Figure 2. Only one trial was rated as low risk of bias, with the remaining eight seen as either high risk or some concerns.

### 3.3 Analysis of Extracted Data

**FIGURE 3:**
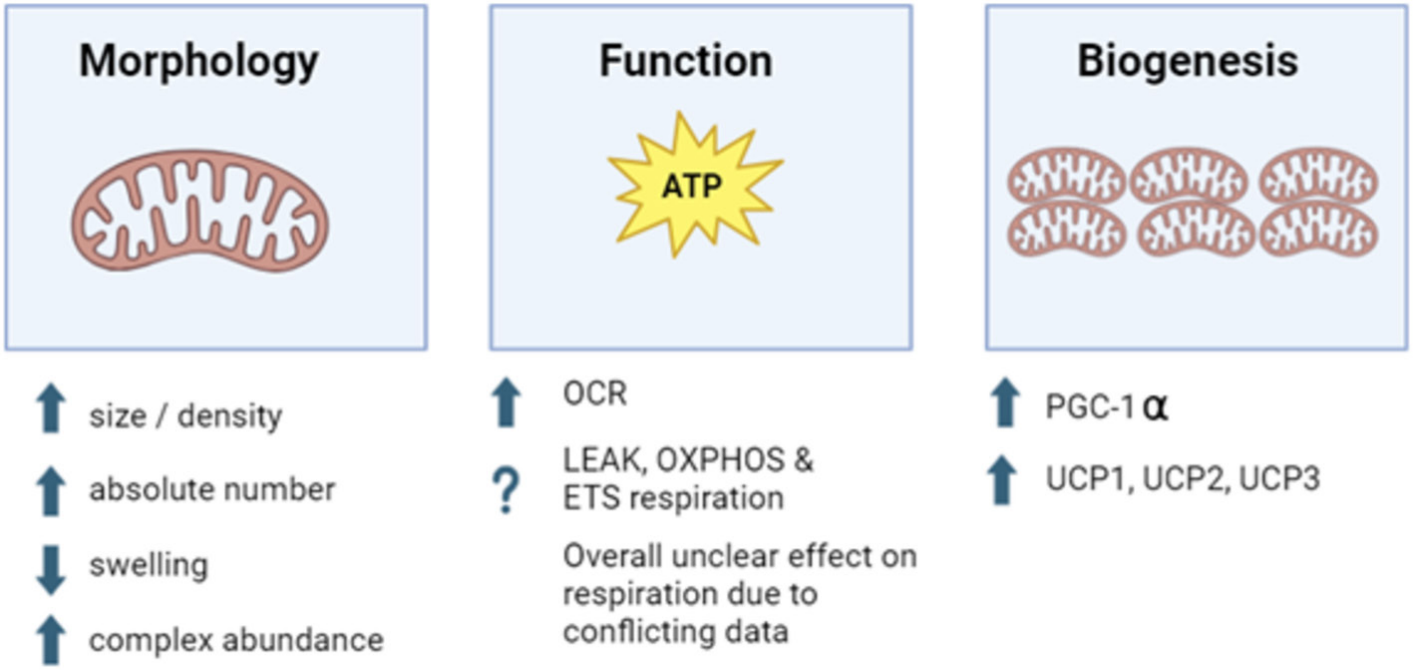
Schematic diagram of the effect of GLP‐1 RA on mitochondria function. Abbreviations: ATP, adenosine triphosphate; ETC, electron transport chain; OCR, oxygen consumption rate; OXPHOS, oxidative phosphorylation; PGC‐1alpha, peroxisome proliferator‐activated receptor gamma coactivator 1‐alpha; UCP, uncoupling protein.

A summary of the results generated by this review can be found in Figure 3. Details can be found in the subsections below.

### 3.4 Mitochondrial Density/Area/Morphology

Ji and colleagues treated KKAy mice with 250 μg/kg/day of liraglutide compared with a saline control. They investigated mitochondrial area and density using transmission electron microscopy (mitochondrial area/100 μm^2^ and mitochondrial number/10 μm^2^ were determined by analysing 10 images taken at 20 000× magnification using image J). They reported a significant increase in mitochondrial area (+113%) and number (+441%) in the liraglutide treated animals compared with the control group [38]. The authors also reported improvements in morphology; The KKAy mouse model (a rodent model of T2DM) presented with disarranged, swollen mitochondria clusters which visually improved following the 6‐week liraglutide treatment. Ren and colleagues treated C57BL/6 mice with semaglutide or a placebo following a high‐fat diet, which resulted in weight gain compared with a group on a normal diet. They used TEM to investigate effects of semaglutide upon mitochondrial number and area. They reported the high fat diet reduced mitochondrial number and area determined by TEM, which was then reversed following semaglutide treatment together with improvements in morphology, that is, reductions in swelling [36]. They also investigated the effect of the drug upon sarcoplasmic reticulum area, but found no difference [36]. Wu and colleagues investigated the effect of exenatide treatment in mice with T2DM upon mitochondria morphology [40]. Using TEM, they found T2DM caused severe disruption to mitochondria morphology, which included swelling and disordered cristae. This was largely reversed after treatment with exenatide leading to the conclusion that the drug was able to reduce mitochondrial damage induced by T2DM.

The effect of liraglutide on mitochondrial mass and complex abundance was investigated in SDT fatty rats (an obese T2DM model) by Yamada and colleagues [37]. They found that increasing doses of liraglutide over 6 weeks increased protein expression of Cox5B (subunit of Complex V) when compared with the control SDT fatty rats [37]. There was also a strong trend for an increase in citrate synthase activity (SDT untreated vs. SDT + liraglutide *p* = 0.05) in the soleus muscle, but no effect was seen in the EDL. CS activity is commonly used as a marker of mitochondrial mass and COX5B was used by the authors to determine mitochondrial preservation.

### 3.5 Mitochondrial Respiration

Mitochondrial respiration was explored in three studies following different GLP‐1 RA therapies. The first study utilized a Seahorse XF24 Extracellular Flux Analyzer to assess oxygen consumption rates (OCR) in C2C12 cells (cells were incubated ± oleate and palmitate acid to mimic obesity) following incubation with 20‐nM Exendin‐4/day for 2 days [43]. Exendin‐4 treatment increased basal and maximal respiration, increased uncoupling efficiency and proton leak compared with untreated cells. The authors commented that this suggested Exendin‐4 promoted an increase in fatty acid oxidation. They also reported an upregulation of UCP1 mRNA after 24 h of 20 nM of Exendin‐4 in differentiated C2C12 cells, which interestingly was not replicated in the animal arm of the study [43]. No effect was seen of exendin treatment on UPC2 or UPC3 mRNA expression. Gao and colleagues noted an increased protein and RNA expression of UCP3 in the EGLP‐1 analogue group when compared with a control and a group treated with Exendin‐4 [42]. Mitochondrial uncoupling proteins are a family of transmembrane proteins localized in the inner mitochondrial membrane that are involved in the transport of protons across the mitochondrial membrane and thereby inducing mitochondrial uncoupling [44]. They are not direct measures of respiratory function but can provide information on thermogenesis and energy expenditure.

Ex vivo mitochondrial respiration using a high‐resolution respirometry via oxygraphy‐2k system (OROBOROS) in C57BL/6Jola HSD mouse models was performed by Jansen and colleagues. High‐fat diet (HFD‐fed) mice were administered 10 μg/kg/day exenatide for 8 weeks. The protocols used specifically probed LEAK and OXPHOS states and electron transfer system capacity [41]. Overall, the authors concluded that mitochondrial respiration was not influenced by exenatide treatment. They did however report that the flux control efficiency showed a significant decrease as well as the maximum complex I activity in HFD‐fed mice supplemented with Exenatide. However, the majority of the LEAK respiration (O2 compensating for the lack of ATP synthesis), OXPHOS respiration (the NADH pathway) and electron transfer system (ETS) respiration were not significantly different when compared with the HFD.

### 3.6 Mitochondrial Biogenesis

C57BL/6J mice given 0.1 mg/kg per day liraglutide for 4 weeks showed an upregulation of PGC‐1α protein expression by 85% in mice on a normal diet and by 204% in those on a high‐fat‐high‐sucrose (HFHS) diet. This effect was independent of effects upon AMPK and SIRT‐1, key proteins within the canonical pathway for increased PGC‐1α expression [39]. However, Yamada and colleagues found no significance in PGC‐1α protein expression following liraglutide in the soleus muscle between the control SD mice and the SDT mice treated with liraglutide, but there was a significantly higher expression of PGC‐1α in the extensor digitorum longus (EDL) muscle [37]. Choung and colleagues [43] reported an increase in PCG‐1α protein expression following incubation of C2C12 cells with 20‐nM Exendin‐4 for 12 h.

## 4 Discussion

This systematic review assessed the current available evidence for the effects of GLP‐1 RA therapy on skeletal muscle respiratory function in people or animal models living with T2DM or obesity. The majority of emerging evidence suggests that, using animal or in vitro models, the administration of GLP‐1 RA has a positive effect upon certain indices of skeletal muscle mitochondrial health. However, this was not a consistent finding, and we do present some unclear and conflicting evidence on mitochondrial mass and PGC‐1α, effects upon which appear to be sensitive to the particular muscle measured [37]. In addition, Jansen and colleagues [41] demonstrated no beneficial effect, that is, a neutral effect on mitochondrial respiration following exenatide administration. Importantly, this study was the only study to measure mitochondrial respiration using high‐resolution respirometry (oxygraphy‐2k system), the gold standard method for this investigation. We also report a complete absence of human studies in this area, making the ability to draw firm conclusions difficult. The majority of trials within this review were deemed as high risk of bias, mainly due to lack of randomization and blinding to outcome measures.

GLP‐1 RA therapy improves glucose levels in people with T2DM by increasing insulin secretion and inhibiting glucagon release. Moreover, GLP‐1 RAs promote satiety and slow gastric emptying, which support weight loss and further improve insulin resistance [45]. Over 4.4 million prescriptions were filled in the United States alone for GLP‐1 RA as of 2023 [46], which reflects the sharp rise in their usage likely due to their efficacy in inducing weight loss and their cardio‐protective effects [47]. Findings from a systematic review and meta‐analyses that assessed weight loss whilst taking liraglutide or exenatide GLP‐1 RA found the range of weight loss from 21 trials ranged from −7.2 to 0.2 kg [48]. However, the authors noted the overall weight loss does not solely consist of fat mass, the growing concern lies on whether there is a negative effect of skeletal muscle loss following GLP‐1 RA therapy [25]. Harder and colleagues found that administration of liraglutide for 8 weeks resulted in a nonsignificant increase in lean body mass in their participants [49], whereas Xaing and colleagues found no significant change in skeletal muscle index but an overall muscle mass loss of 4.8 ± 4.4% in Chinese people with obesity following semaglutide treatment for 6 months [50]. Importantly, data from clinical populations suggest that GLP‐1 RA therapy may actually result in muscle atrophy as dulaglutide was reported to have a significantly negative effect on skeletal muscle mass in people with T2DM on haemodialysis. The authors concluded, therefore, that in haemodialysis patients, an already very frail population, GLP‐1 RA may actually promote sarcopenia [51]. This suggests that the effect of GLP‐1 RA on skeletal muscle may vary between populations, which is critical to understand. The clinical relevance of these negative effects on muscle is still up for debate as similar losses in muscle have been found from general weight loss making the need for further research vital in understanding the overall effect [52].

With data being limited, and no consensus on the overall effect upon skeletal muscle mass, a clear understanding of the mechanisms at play is yet to be determined. Exercise plays a key role in the preservation of skeletal muscle in age and chronic disease, but not all individuals can meet the daily exercise recommendations [53]. There are some suggestions that GLP‐1 RA therapy itself may support improvements in physical function [54]. Weight loss interventions usually involve a combination of dietetic interventions and physical activity, with the inclusion of pharmacological therapies as a recent addition to weight management strategies. Therefore, more data are needed here to fully understand the interaction of this drug and lifestyle modifications on skeletal muscle mass in different populations. Exercise is well known for improving mitochondrial health [55], and improvements in mitochondria are vital in maintaining skeletal muscle function due to their role in energy homeostasis and metabolism [56]. By understanding what effect GLP‐1 RA has on mitochondria, we can then begin to understand the overall effect it may have on skeletal muscle mass. It is possible that these effects are conflicting in that GLP‐1s have a positive impact upon mitochondrial content or structure, but a negative effect upon muscle mass.

Studies show that stopping GLP‐1 RA treatment often leads to significant weight regain, with up to two thirds of lost weight regained within a year, along with reversals in some cardiometabolic improvements [57]. These findings emphasize the need for ongoing GLP‐1 RAs treatment to maintain weight loss and sustain cardiometabolic benefits, but it is also important to understand the impact of weight regain on mitochondrial content/function, particularly because most regained weight may be fat mass rather than muscle mass [58, 59]. Notably, none of the studies reviewed included a follow‐up period post treatment, highlighting an area for future research.

### 4.1 Mitochondrial Morphology

Overall, the studies analysed showed a beneficial effect of GLP‐1 RA therapy upon mitochondria morphology. This included increased size and density [38] [36], [40] increased absolute numbers/content [36, 37, 38], reduction in signs of mitochondrial swelling [40] and complex abundance [37]. Such improvements are vital for efficient mitochondrial function. An increase in mitochondrial size and density improves skeletal muscle oxidative capacity, which has been linked to increasing levels of resting metabolic rate (BMR) [60] supporting an overall increase in BMR. This increase in BMR could help support people who are prescribed GLP‐1 RA with their overall weight loss effect due to the natural decrease in BMR as people lose weight [61]. Ren and colleagues noticed increased mitochondrial content following 12 weeks of semaglutide treatment [36]—increased mitochondria content is correlated to improvements in skeletal muscle function and health [62]. Similarly, Ji and colleagues also saw increases in mitochondria number/content and area along with improvements within the myofibrils structure following liraglutide treatment (clearer cross striation and less atrophic myofibril compared with the mice with diabetes) [38]. These improvements show potential for liraglutide to protect and repair damaged mitochondria through decreasing oxidative stress, which is generated by dysfunctional mitochondria [63]. Exenatide was also shown to improve mitochondrial morphology after 8 weeks in rats with T2DM, with a smaller number of swollen mitochondria found when compared with the control T2DM rat without exenatide treatment [40]. This decrease allows for better ATP production and utilization within the muscle, which can support in the maintenance of skeletal muscle structure and integrity [64].

Mitochondria content improvements were noted by increased CS and upregulated Cox5B in the soleus muscle of SDT rats following liraglutide treatment for 8 weeks [37]. This study demonstrates the possible role of liraglutide in upregulation of fatty acid oxidation by preserving mitochondria content in type I muscle fibres [65]. This preservation can reduce the risk of mitochondrial dysfunction and cell death, helping to maintain the skeletal muscle integrity in patients.

It is important that researchers combine measures of mitochondrial content and function, as one does not always follow the other (i.e., increased content does not always lead to increased function), and careful consideration should be made to the different types of mitochondria function beyond ATP synthesis.

### 4.2 Mitochondrial Respiration

There is no clear overall effect of GLP‐1 RA on mitochondrial respiration. Choung and colleagues used the Seahorse XF24 extracellular Flux analyser to explore the effects of Exendin‐4 on C2C12 cells, which were given palmitate to induce obesity‐like properties [43]. OCR, basal respiration rate, and proton leak where all seen to increase following the Exendin‐4 treatment; there was also an increase in UCP1 expression, which can correlate with the increased energy metabolism [66]. A similar upregulation of UCP2 or UCP3 though was not reported [43], whereas Gao and colleagues did report upregulation of UCP2 and UCP3 following EGLP‐1 analogue treatment when compared with Exendin‐4 treatment in C2C12 cells [42]. UCP2 regulation has been noted to support ATP/ADP ratio, whereas UCP3 is involved in regulation of ROS and the handling of fatty acids [67]. These changes following GLP‐1 therapy may protect the mitochondria from overaccumulation of fatty acids, which may play a part in reducing skeletal muscle insulin resistance [68].

High‐resolution respirometry was used to understand the effects of exenatide on mitochondrial respiration in HFD‐fed mice [41]. There was deemed no significant overall effect as there was no difference reported in LEAK, OXPHOS or ETS respiration in the treatment mice compared with the control HFD mice. They did however report a decrease in both flux control efficiency and maximum complex I activity [41], which have been noted to improve glucose homeostasis [69]. This is the only known study to date that has investigated mitochondrial respiration using high‐resolution respirometry in skeletal muscle following GLP‐1 RA therapy. It is, therefore, still unclear if there is any benefit of GLP‐1 RA's on mitochondrial respiration health.

### 4.3 Mitochondria Biogenesis

Two papers focused on the expression of PGC‐1α [37, 39]. Yamanda and colleagues noted an upregulation of PGC‐1α expression in SDT rats EDL muscle following liraglutide treatment, but not in the soleus muscle [37], which is interesting given the predominance of type I fibres within the soleus muscle [70]. Protein expression of PGC‐1α was also shown to be upregulated in HFHS mice given liraglutide over 4 weeks by Zhou and colleagues; however, clear indication of which muscle was used to find this expression was not given. This upregulation was not mediated through the AMPK‐SIRT‐1 cell signalling pathway, although it shows signs of supporting mitochondria capacity through other pathways, which need further investigation [39]. The authors also reported an increase in PGC‐1α protein expression in the control + liraglutide compared with the control group alone [39]. Zhou and colleagues did not report on the relationship between PGC‐1α and mitochondrial content present, which would have been beneficial in showing a clearer understanding of the effects of GLP‐1 RA. PGC‐1α is a critical regulator in mitochondria biogenesis, and its expression is often used as an indicator of the activation of this process. In addition to this, it supports quality control mechanisms within mitochondria through fission, mitophagy and fusion [71]. Therefore, determining the effects of GLP‐1 therapy on PGC‐1α are a vital component to help us understand the effects this class of drugs might be having upon on key aspects of mitochondria health [71].

### 4.4 Limitations

The biggest limitation to the current available data is the lack of human studies that have been performed to investigate skeletal muscle respiratory function following GLP‐1 RA–based therapies. This is a major gap within the current research that is vital in understanding the effects of GLP‐1 RA–based therapies on skeletal muscle. The effect of weight loss drugs in general upon skeletal muscle is an emerging area of research clearly at an early stage, which likely accounts for the absence of human data. Future studies should include assessments of respiratory function, which can be made by magnetic resonance spectroscopy (MRS) to avoid the necessity for muscle biopsies in this population. This need for human research is also emphasized by the poor translation of drug trials from animal to human models [72]. In addition, different weight management therapies may influence mitochondria in different ways; a meta‐analysis on the mitochondrial effects comparing caloric restriction (CR) and bariatric surgery found a reduction of complex IV in CR group but not in the bariatric group [73]. There is no clear understanding on whether the effects of GLP‐1 RA on mitochondria are caused by the drug or due to weight‐independent mechanisms, which will need further evaluation. Furthermore, this review only found data on the effect of single GLP‐1 RA's on mitochondrial respiration. As multiple novel pharmacotherapies combine GLP‐1 with other enteropancreatic hormones with diverse actions as dual or triple agonists, it is important to understand over the next years the effect of the different treatment combinations with GLP‐1 on mitochondria.

There is a lack of studies looking at the impact of exercise alongside GLP‐1 RA administration on skeletal muscle, which is vital because of the key role exercise plays in weight management as well as the role mitochondria plays in exercise. A study by Yates and colleagues found a decrease in physical activity following GLP‐1 RA therapy despite the overall decline in weight loss [74], yet there is a positive effect on physical function [54]. Reasoning for this is conflicting relation is limited and warrants further attention towards the involvement of exercise during administration. It is also important to understand what effects persist when individuals stop using GLP‐1 RAs. For example, it is not known how long improvements in physical function or mitochondrial content will persist after stopping GLP‐1 RAs and whether potential changes in these parameters will be linked to weight regain after stopping GLP‐1 RAs.

Because of differences in the drug therapy, dosages, outcome measures, duration of application and model used, a meta‐analysis could not be completed and so an overall effect cannot be determined based on current data available. Only one trial was considered as low risk of bias, as majority of papers did not blind the outcome reporter to the groups or failed to report their randomization/blinding procedure if applicable. None of the studies included have reported direct number statistics in their results, leaving interpretation difficult due to the only values given being through bar graphs or their own interpretation of the results. A standardization of dosage levels could help to untangle the relative effect as the studies assessed here did not present a rationale for their dosage levels or their prior optimization. Furthermore, how the dosages used within preclinical trials were selected or how they relate to those administered to humans was not discussed in any study we evaluated. This is a limitation as it puts into question the degree of translation in the effects seen in these animal models and what might be expected to be seen in humans.

## 5 Conclusion

The current data presented through this systematic review demonstrates the potential beneficial effects of GLP‐1 RA therapy on mitochondrial content with an unclear effect upon function and mass. However, data are very limited and solely presented in animal models. Future studies should be conducted in human populations in order to begin to understand the effect of GLP‐1 RAs on human skeletal muscle mitochondria. Finally, skeletal muscle responds to stressors in a fibre type specific manner. Therefore, future investigations should seek to determine the effects of GLP‐1 RAs on the different muscle fibre types. Treating muscle as a homogenous tissue may mean that important differences are missed.

## Ethics Statement

We certify that we comply with the ethical guidelines for authorship and publishing.

## Conflicts of Interest

The authors declare no conflicts of interest.

## References

[1] H. A. Afolabi , Z. Zakariya , A. B. Ahmed Shokri , et al., “The Relationship Between Obesity and Other Medical Comorbidities,” Obesity Medicine 17 (2020): 100164.

[2] M. A. B. Khan , M. J. Hashim , J. K. King , R. D. Govender , H. Mustafa , and J. al Kaabi , “Epidemiology of Type 2 Diabetes—Global Burden of Disease and Forecasted Trends,” Journal of Epidemiology and Global Health 10, no. 1 (2020): 107–111. 32175717 10.2991/jegh.k.191028.001 PMC7310804

[3] J. P. H. Wilding , “The Importance of Weight Management in Type 2 Diabetes Mellitus,” International Journal of Clinical Practice 68, no. 6 (2014): 682–691. 24548654 10.1111/ijcp.12384 PMC4238418

[4] J. Blahova , M. Martiniakova , M. Babikova , V. Kovacova , V. Mondockova , and R. Omelka , “Pharmaceutical Drugs and Natural Therapeutic Products for the Treatment of Type 2 Diabetes Mellitus,” Pharmaceuticals (Basel) 14, no. 8 (2021): 806. 34451903 10.3390/ph14080806 PMC8398612

[5] M. B. Davidson , G. Bate , and P. Kirkpatrick , “Exenatide,” Nature Reviews. Drug Discovery 4, no. 9 (2005): 713–714. 10.1038/nrd1828 16178120

[6] O. Dzaye , P. Berning , A. C. Razavi , et al., “Online Searches for SGLT‐2 Inhibitors and GLP‐1 Receptor Agonists Correlate With Prescription Rates in the United States: An Infodemiological Study,” Frontiers in Cardiovascular Medicine 9 (2022): 936651. 35966558 10.3389/fcvm.2022.936651 PMC9372305

[7] X. Pi‐Sunyer , A. Astrup , K. Fujioka , et al., “A Randomized, Controlled Trial of 3.0 mg of Liraglutide in Weight Management,” New England Journal of Medicine 373, no. 1 (2015): 11–22. 26132939 10.1056/NEJMoa1411892

[8] M. J. Davies , R. Bergenstal , B. Bode , et al., “Efficacy of Liraglutide for Weight Loss Among Patients With Type 2 Diabetes: The SCALE Diabetes Randomized Clinical Trial,” JAMA 314, no. 7 (2015): 687–699. 26284720 10.1001/jama.2015.9676

[9] M. Davies , L. Færch , O. K. Jeppesen , et al., “Semaglutide 2·4 mg Once a Week in Adults With Overweight or Obesity, and Type 2 Diabetes (STEP 2): A Randomised, Double‐Blind, Double‐Dummy, Placebo‐Controlled, Phase 3 Trial,” Lancet 397, no. 10278 (2021): 971–984. 33667417 10.1016/S0140-6736(21)00213-0

[10] J. P. H. Wilding , R. L. Batterham , S. Calanna , et al., “Once‐Weekly Semaglutide in Adults With Overweight or Obesity,” New England Journal of Medicine 384, no. 11 (2021): 989–1002. 33567185 10.1056/NEJMoa2032183

[11] E. Melson , U. Ashraf , D. Papamargaritis , and M. J. Davies , “What Is the Pipeline for Future Medications for Obesity?,” International Journal of Obesity (2024), 10.1038/s41366-024-01473-y. PMC11971045 38302593

[12] A. M. Jastreboff , L. J. Aronne , N. N. Ahmad , et al., “Tirzepatide Once Weekly for the Treatment of Obesity,” New England Journal of Medicine 387, no. 3 (2022): 205–216. 35658024 10.1056/NEJMoa2206038

[13] W. T. Garvey , J. P. Frias , A. M. Jastreboff , et al., “Tirzepatide Once Weekly for the Treatment of Obesity in People With Type 2 Diabetes (SURMOUNT‐2): A Double‐Blind, Randomised, Multicentre, Placebo‐Controlled, Phase 3 Trial,” Lancet 402, no. 10402 (2023): 613–626. 37385275 10.1016/S0140-6736(23)01200-X

[14] N. Sattar , M. M. Y. Lee , S. L. Kristensen , et al., “Cardiovascular, Mortality, and Kidney Outcomes With GLP‐1 Receptor Agonists in Patients With Type 2 Diabetes: A Systematic Review and Meta‐Analysis of Randomised Trials,” Lancet Diabetes and Endocrinology 9, no. 10 (2021): 653–662. 34425083 10.1016/S2213-8587(21)00203-5

[15] V. Perkovic , K. R. Tuttle , P. Rossing , et al., “Effects of Semaglutide on Chronic Kidney Disease in Patients With Type 2 Diabetes,” New England Journal of Medicine 391, no. 2 (2024): 109–121. 38785209 10.1056/NEJMoa2403347

[16] A. M. Lincoff , K. Brown‐Frandsen , H. M. Colhoun , et al., “Semaglutide and Cardiovascular Outcomes in Obesity Without Diabetes,” New England Journal of Medicine 389, no. 24 (2023): 2221–2232. 37952131 10.1056/NEJMoa2307563

[17] X. H. Pan , B. Tan , Y. H. Chin , et al., “Efficacy and Safety of Tirzepatide, GLP‐1 Receptor Agonists, and Other Weight Loss Drugs in Overweight and Obesity: A Network Meta‐Analysis,” Obesity (Silver Spring) 32, no. 5 (2024): 840–856. 38413012 10.1002/oby.24002

[18] M. N. Kosiborod , S. Z. Abildstrøm , B. A. Borlaug , et al., “Semaglutide in Patients With Heart Failure With Preserved Ejection Fraction and Obesity,” New England Journal of Medicine 389, no. 12 (2023): 1069–1084. 37622681 10.1056/NEJMoa2306963

[19] M. N. Kosiborod , M. C. Petrie , B. A. Borlaug , et al., “Semaglutide in Patients With Obesity‐Related Heart Failure and Type 2 Diabetes,” New England Journal of Medicine 390, no. 15 (2024): 1394–1407. 38587233 10.1056/NEJMoa2313917

[20] R. Loomba , M. L. Hartman , E. J. Lawitz , et al., “Tirzepatide for Metabolic Dysfunction‐Associated Steatohepatitis With Liver Fibrosis,” New England Journal of Medicine 391, no. 4 (2024): 299–310. 38856224 10.1056/NEJMoa2401943

[21] T. Cukierman‐Yaffe , H. C. Gerstein , H. M. Colhoun , et al., “Effect of Dulaglutide on Cognitive Impairment in Type 2 Diabetes: An Exploratory Analysis of the REWIND Trial,” Lancet Neurology 19, no. 7 (2020): 582–590. 32562683 10.1016/S1474-4422(20)30173-3

[22] C. H. Nørgaard , S. Friedrich , C. T. Hansen , et al., “Treatment With Glucagon‐Like Peptide‐1 Receptor Agonists and Incidence of Dementia: Data From Pooled Double‐Blind Randomized Controlled Trials and Nationwide Disease and Prescription Registers,” Alzheimer's & Dementia: Translational Research & Clinical Interventions 8, no. 1 (2022): e12268. 35229024 10.1002/trc2.12268 PMC8864443

[23] W. G. Meissner , P. Remy , C. Giordana , et al., “Trial of Lixisenatide in Early Parkinson's Disease,” New England Journal of Medicine 390, no. 13 (2024): 1176–1185. 38598572 10.1056/NEJMoa2312323

[24] L. Wang , R. Xu , D. C. Kaelber , and N. A. Berger , “Glucagon‐Like Peptide 1 Receptor Agonists and 13 Obesity‐Associated Cancers in Patients With Type 2 Diabetes,” JAMA Network Open 7, no. 7 (2024): e2421305. 38967919 10.1001/jamanetworkopen.2024.21305 PMC11227080

[25] J. A. Sargeant , J. Henson , J. A. King , T. Yates , K. Khunti , and M. J. Davies , “A Review of the Effects of Glucagon‐Like Peptide‐1 Receptor Agonists and Sodium‐Glucose Cotransporter 2 Inhibitors on Lean Body Mass in Humans,” Endocrinology and Metabolism 34, no. 3 (2019): 247–262. 31565876 10.3803/EnM.2019.34.3.247 PMC6769337

[26] M. Tieland , I. Trouwborst , and B. C. Clark , “Skeletal Muscle Performance and Ageing,” Journal of Cachexia, Sarcopenia and Muscle 9, no. 1 (2018): 3–19. 29151281 10.1002/jcsm.12238 PMC5803609

[27] E. Ahmad , J. A. Sargeant , T. Yates , D. R. Webb , and M. J. Davies , “Type 2 Diabetes and Impaired Physical Function: A Growing Problem,” Diabetology 3, no. 1 (2022): 30–45.

[28] S. B. K. Jensen , V. Sørensen , R. M. Sandsdal , et al., “Bone Health After Exercise Alone, GLP‐1 Receptor Agonist Treatment, or Combination Treatment: A Secondary Analysis of a Randomized Clinical Trial,” JAMA Network Open 7, no. 6 (2024): e2416775. 38916894 10.1001/jamanetworkopen.2024.16775 PMC11200146

[29] I. Daniilopoulou , E. Vlachou , G. I. Lambrou , et al., “The Impact of GLP1 Agonists on Bone Metabolism: A Systematic Review,” Medicina (Kaunas, Lithuania) 58, no. 2 (2022): 224. 35208548 10.3390/medicina58020224 PMC8878541

[30] C. A. Pileggi , B. G. Hooks , R. McPherson , R. R. M. Dent , and M. E. Harper , “Targeting Skeletal Muscle Mitochondrial Health in Obesity,” Clinical Science (London, England) 136, no. 14 (2022): 1081–1110. 10.1042/CS20210506 PMC9334731 35892309

[31] S. Rovira‐Llopis , C. Bañuls , N. Diaz‐Morales , A. Hernandez‐Mijares , M. Rocha , and V. M. Victor , “Mitochondrial Dynamics in Type 2 Diabetes: Pathophysiological Implications,” Redox Biology 11 (2017): 637–645. 28131082 10.1016/j.redox.2017.01.013 PMC5284490

[32] M. Gonzalez‐Freire , F. Adelnia , R. Moaddel , and L. Ferrucci , “Searching for a Mitochondrial Root to the Decline in Muscle Function With Ageing,” Journal of Cachexia, Sarcopenia and Muscle 9, no. 3 (2018): 435–440. 29774990 10.1002/jcsm.12313 PMC5989834

[33] J. L. Gamboa , B. Roshanravan , T. Towse , et al., “Skeletal Muscle Mitochondrial Dysfunction Is Present in Patients With CKD Before Initiation of Maintenance Hemodialysis,” Clinical Journal of the American Society of Nephrology 15, no. 7 (2020): 926–936. 32591419 10.2215/CJN.10320819 PMC7341789

[34] D. E. Kelley , J. He , E. V. Menshikova , and V. B. Ritov , “Dysfunction of Mitochondria in Human Skeletal Muscle in Type 2 Diabetes,” Diabetes 51 (2002): 2944–2950. 12351431 10.2337/diabetes.51.10.2944

[35] D. Moher , A. Liberati , J. Tetzlaff , D. G. Altman , and Prisma Group , “Preferred Reporting Items for Systematic Reviews and Meta‐Analyses: The PRISMA Statement,” PLoS Medicine 6, no. 6 (2009): e1000097. 19621072 10.1371/journal.pmed.1000097 PMC2707599

[36] Q. Ren , S. Chen , X. Chen , et al., “An Effective Glucagon‐Like Peptide‐1 Receptor Agonists, Semaglutide, Improves Sarcopenic Obesity in Obese Mice by Modulating Skeletal Muscle Metabolism,” Drug Design, Development and Therapy 16 (2022): 3723–3735. 36304787 10.2147/DDDT.S381546 PMC9594960

[37] S. Yamada , Y. Ogura , K. Inoue , et al., “Effect of GLP‐1 Receptor Agonist, Liraglutide, on Muscle in Spontaneously Diabetic Torii Fatty Rats,” Molecular and Cellular Endocrinology 539 (2022): 111472. 34606964 10.1016/j.mce.2021.111472

[38] W. Ji , X. Chen , J. Lv , et al., “Liraglutide Exerts Antidiabetic Effect via PTP1B and PI3K/Akt2 Signaling Pathway in Skeletal Muscle of KKAy Mice,” International Journal of Endocrinology 2014 (2014): 312452. 25183970 10.1155/2014/312452 PMC4144308

[39] J. Zhou , A. Poudel , P. Chandramani‐Shivalingappa , B. Xu , R. Welchko , and L. Li , “Liraglutide Induces Beige Fat Development and Promotes Mitochondrial Function in Diet Induced Obesity Mice Partially Through AMPK‐SIRT‐1‐PGC1‐Alpha Cell Signaling Pathway,” Endocrine 64, no. 2 (2019): 271–283. 30535743 10.1007/s12020-018-1826-7

[40] H. Wu , C. Sui , F. Xia , et al., “Effects of Exenatide Therapy on Insulin Resistance in the Skeletal Muscles of High‐Fat Diet and Low‐Dose Streptozotocin‐Induced Diabetic Rats,” Endocrine Research 41, no. 1 (2016): 1–7. 26361069 10.3109/07435800.2015.1015726

[41] K. M. Jansen , N. Dahdah , P. Gama‐Perez , P. C. Schots , T. S. Larsen , and P. M. Garcia‐Roves , “Impact of GLP‐1 Receptor Agonist Versus Omega‐3 Fatty Acids Supplement on Obesity‐Induced Alterations of Mitochondrial Respiration,” Frontiers in Endocrinology 14 (2023): 1098391. 37033212 10.3389/fendo.2023.1098391 PMC10076843

[42] H. Gao , Q. Zhao , K. Li , et al., “EGLP‐1 Lowers Body Weight Better Than Exendin‐4 by Reducing Food Intake and Increasing Basal Energy Expenditure in Diet‐Induced Obese Mice,” Experimental Cell Research 399, no. 1 (2021): 112454. 33359447 10.1016/j.yexcr.2020.112454

[43] J. S. Choung , Y. S. Lee , and H. S. Jun , “Exendin‐4 Increases Oxygen Consumption and Thermogenic Gene Expression in Muscle Cells,” Journal of Molecular Endocrinology 58, no. 2 (2017): 79–90. 27872157 10.1530/JME-16-0078

[44] S. Demine , P. Renard , and T. Arnould , “Mitochondrial Uncoupling: A key Controller of Biological Processes in Physiology and Diseases,” Cells 8, no. 8 (2019): 795. 31366145 10.3390/cells8080795 PMC6721602

[45] D. J. Drucker , “Mechanisms of Action and Therapeutic Application of Glucagon‐Like Peptide‐1,” Cell Metabolism 27, no. 4 (2018): 740–756. 29617641 10.1016/j.cmet.2018.03.001

[46] E. Mahase , “GLP‐1 Agonist Shortage Will Last Until end of 2024, Government Warns,” BMJ 384 (2024): q28. 38182266 10.1136/bmj.q28

[47] S. P. Marso , S. C. Bain , A. Consoli , et al., “Semaglutide and Cardiovascular Outcomes in Patients With Type 2 Diabetes,” New England Journal of Medicine 375, no. 19 (2016): 1834–1844. 27633186 10.1056/NEJMoa1607141

[48] T. Vilsbøll , M. Christensen , A. E. Junker , F. K. Knop , and L. L. Gluud , “Effects of Glucagon‐Like Peptide‐1 Receptor Agonists on Weight Loss: Systematic Review and Meta‐Analyses of Randomised Controlled Trials,” BMJ 344 (2012): d7771. 22236411 10.1136/bmj.d7771 PMC3256253

[49] H. Harder , L. Nielsen , T. D. T. Thi , and A. Astrup , “The Effect of Liraglutide, a Long‐Acting Glucagon‐Like Peptide 1 Derivative, on Glycemic Control, Body Composition, and 24‐H Energy Expenditure in Patients With Type 2 Diabetes,” Diabetes Care 27, no. 8 (2004): 1915–1921. 15277417 10.2337/diacare.27.8.1915

[50] J. Xiang , X. Y. Ding , W. Zhang , et al., “Clinical Effectiveness of Semaglutide on Weight Loss, Body Composition, and Muscle Strength in Chinese Adults,” European Review for Medical and Pharmacological Sciences 27, no. 20 (2023): 9908–9915. 37916360 10.26355/eurrev_202310_34169

[51] T. Yajima , K. Yajima , H. Takahashi , and K. Yasuda , “The Effect of Dulaglutide on Body Composition in Type 2 Diabetes Mellitus Patients on Hemodialysis,” Journal of Diabetes and its Complications 32, no. 8 (2018): 759–763. 29937137 10.1016/j.jdiacomp.2018.05.018

[52] C. Conte , K. D. Hall , and S. Klein , “Is Weight Loss–Induced Muscle Mass Loss Clinically Relevant?,” JAMA 332 (2024): 9. 38829659 10.1001/jama.2024.6586

[53] G. Distefano and B. H. Goodpaster , “Effects of Exercise and Aging on Skeletal Muscle,” Cold Spring Harbor Perspectives in Medicine 8, no. 3 (2018): a029785. 28432116 10.1101/cshperspect.a029785 PMC5830901

[54] E. Ahmad , F. Arsenyadis , A. Almaqhawi , et al., “Impact of Novel Glucose‐Lowering Therapies on Physical Function in People With Type 2 Diabetes: A Systematic Review and Meta‐Analysis of Randomised Placebo‐Controlled Trials,” Diabetic Medicine 40, no. 6 (2023): e15083. 36905324 10.1111/dme.15083

[55] J. M. Memme , A. T. Erlich , G. Phukan , and D. A. Hood , “Exercise and Mitochondrial Health,” Journal of Physiology 599, no. 3 (2021): 803–817. 31674658 10.1113/JP278853

[56] I. R. Lanza and K. Sreekumaran Nair , “Regulation of Skeletal Muscle Mitochondrial Function: Genes to Proteins,” Acta Physiologica 199, no. 4 (2010): 529–547. 20345409 10.1111/j.1748-1716.2010.02124.x PMC3070482

[57] J. P. H. Wilding , R. L. Batterham , M. Davies , et al., “Weight Regain and Cardiometabolic Effects After Withdrawal of Semaglutide: The STEP 1 Trial Extension,” Diabetes, Obesity & Metabolism 24, no. 8 (2022): 1553–1564. 10.1111/dom.14725 PMC9542252 35441470

[58] S. B. K. Jensen , M. B. Blond , R. M. Sandsdal , et al., “Healthy Weight Loss Maintenance With Exercise, GLP‐1 Receptor Agonist, or Both Combined Followed by One Year Without Treatment: A Post‐Treatment Analysis of a Randomised Placebo‐Controlled Trial,” EClinicalMedicine 69 (2024): 102475. 38544798 10.1016/j.eclinm.2024.102475 PMC10965408

[59] T. Yates , G. J. H. Biddle , J. Henson , et al., “Impact of Weight Loss and Weight Gain Trajectories on Body Composition in a Population at High Risk of Type 2 Diabetes: A Prospective Cohort Analysis,” Diabetes, Obesity & Metabolism 26, no. 3 (2024): 1008–1015. 10.1111/dom.15400 38093678

[60] M. Zampino , R. D. Semba , F. Adelnia , et al., “Greater Skeletal Muscle Oxidative Capacity Is Associated With Higher Resting Metabolic Rate: Results From the Baltimore Longitudinal Study of Aging,” Journals of Gerontology. Series A, Biological Sciences and Medical Sciences 75, no. 12 (2020): 2262–2268. 32201887 10.1093/gerona/glaa071 PMC7751004

[61] R. L. Leibel , M. Rosenbaum , and J. Hirsch , “Changes in Energy Expenditure Resulting From Altered Body Weight,” New England Journal of Medicine 332, no. 10 (1995): 621–628. 7632212 10.1056/NEJM199503093321001

[62] G. Swalsingh , P. Pani , and N. C. Bal , “Structural Functionality of Skeletal Muscle Mitochondria and Its Correlation With Metabolic Diseases,” Clinical Science 136, no. 24 (2022): 1851–1871. 36545931 10.1042/CS20220636

[63] C. Guo , L. Sun , X. Chen , and D. Zhang , “Oxidative Stress, Mitochondrial Damage and Neurodegenerative Diseases,” Neural Regeneration Research 8, no. 21 (2013): 2003–2014. 25206509 10.3969/j.issn.1673-5374.2013.21.009 PMC4145906

[64] Y. Potes , Z. Pérez‐Martinez , J. C. Bermejo‐Millo , et al., “Overweight in the Elderly Induces a Switch in Energy Metabolism That Undermines Muscle Integrity,” Aging and Disease 10, no. 2 (2019): 217–230. 31011474 10.14336/AD.2018.0430 PMC6457058

[65] H. Dong and S. Y. Tsai , “Mitochondrial Properties in Skeletal Muscle Fiber,” Cells 12, no. 17 (2023): 2183. 37681915 10.3390/cells12172183 PMC10486962

[66] R. A. Busiello , S. Savarese , and A. Lombardi , “Mitochondrial Uncoupling Proteins and Energy Metabolism,” Frontiers in Physiology 6 (2015): 36. 25713540 10.3389/fphys.2015.00036 PMC4322621

[67] P. Schrauwen and M. Hesselink , “UCP2 and UCP3 in Muscle Controlling Body Metabolism,” Journal of Experimental Biology 205, no. Pt 15 (2002): 2275–2285. 12110661 10.1242/jeb.205.15.2275

[68] A. R. Martins , R. T. Nachbar , R. Gorjao , et al., “Mechanisms Underlying Skeletal Muscle Insulin Resistance Induced by Fatty Acids: Importance of the Mitochondrial Function,” Lipids in Health and Disease 11 (2012): 30. 22360800 10.1186/1476-511X-11-30 PMC3312873

[69] W. L. Hou , J. Yin , M. Alimujiang , et al., “Inhibition of Mitochondrial Complex I Improves Glucose Metabolism Independently of AMPK Activation,” Journal of Cellular and Molecular Medicine 22, no. 2 (2018): 1316–1328. 29106036 10.1111/jcmm.13432 PMC5783883

[70] P. D. Gollnick , B. Sjödin , J. Karlsson , E. Jansson , and B. Saltin , “Human Soleus Muscle: A Comparison of Fiber Composition and Enzyme Activities With Other leg Muscles,” Pflügers Archiv 348, no. 3 (1974): 247–255. 4275915 10.1007/BF00587415

[71] J. F. Halling and H. Pilegaard , “PGC‐1α‐Mediated Regulation of Mitochondrial Function and Physiological Implications,” Applied Physiology, Nutrition, and Metabolism 45, no. 9 (2020): 927–936. 10.1139/apnm-2020-0005 32516539

[72] L. J. Marshall , J. Bailey , M. Cassotta , K. Herrmann , and F. Pistollato , “Poor Translatability of Biomedical Research Using Animals—A Narrative Review,” Alternatives to Laboratory Animals 51, no. 2 (2023): 102–135. 36883244 10.1177/02611929231157756

[73] M. Pérez‐Rodríguez , J. R. Huertas , J. M. Villalba , and R. A. Casuso , “Mitochondrial Adaptations to Calorie Restriction and Bariatric Surgery in Human Skeletal Muscle: A Systematic Review With Meta‐Analysis,” Metabolism 138 (2023): 155336. 36302454 10.1016/j.metabol.2022.155336

[74] T. Yates , J. A. Sargeant , J. A. King , et al., “Initiation of New Glucose‐Lowering Therapies May Act to Reduce Physical Activity Levels: Pooled Analysis From Three Randomized Trials,” Diabetes Care 45, no. 11 (2022): 2749–2752. 35984425 10.2337/dc22-0888

